# The Proposed Constitution and By-Laws for County Societies

**Published:** 1902-09

**Authors:** 


### EDITORIAL NOTES AND COMMENTS.

The Business office of THE JOURNAL-RECORD is No. 64 Marietta Street.

The Editorial office is 318-319-320 The Prudential.

Address all Business communications to Dr. M. B. Hutchins, Mgr.

Make remittances payable to THE ATLANTA JOURNAL-RECORD OF MEDICINE.

On matters pertaining to the Editorial and Original Communications address Dr. Bernard Wolff, Atlanta.

Reprints of original articles will be furnished at cost price. Requests for the same should always be made *on the manuscript*.

We will present, post-paid, on request, to each contributor of an original article, twenty (20) marked copies of THE JOURNAL-RECORD containing such article.

### THE PROPOSED CONSTITUTION AND BY-LAWS FOR COUNTY SOCIETIES.

THE committee appointed by the American Medical Association to prepare a constitution and by-laws for county societies in conformity with and in confirmation of the general plan of organization already adopted for the association and State societies, has offered the following which we give in full and which merits very careful consideration. The report is suggestive rather than mandatory. It is intended as a model and is capable of such local modification and emendations as may seem fit in special instances without losing sight of the main intent, to bring about solidarity in the medical profession :

### CONSTITUTION.

#### ARTICLE I.—NAME AND TITLE OF THE SOCIETY.

The name and title of this organization shall be the ———— County Medical Society.

#### ARTICLE II.—PURPOSES OF THE SOCIETY.

The purposes of this Society shall be to bring into one organization the physicians of this county; and by frequent meetings and full and frank interchange of views to secure such intelligent unity and harmony in every phase of their labor as will elevate and effectuate the opinions of the profession in all scientific, legislative, public health, material and social affairs, to the end that it may receive that respect and support within its own ranks and from the community to which its honorable history and great achievements entitle it; and with other county societies to form the ———— State Medical Association, and through it, with other State associations, to form and maintain the American Medical Association.

ARTICLE III.—COMPOSITION.

Every physician residing and practicing in ———— and legally registering as such, and who is in good professional standing, shall be eligible for membership.

ARTICLE IV.—MEETING.

Regular meetings shall be held monthly (or oftener) at such time and place as may be determined by the Society.

ARTICLE V.—OFFICERS.

The officers of this Society shall consist of a President, Vice-President, Secretary, Treasurer and Delegates. These officers, except the Delegates, who shall be elected for two years, shall be elected annually for a term of one year, and until their successors are elected and installed.

ARTICLE VI.—FUNDS AND EXPENSES.

Funds for meeting the expenses of the Society shall be raised by annual dues, special assessments and voluntary contribution. Funds may be appropriated by vote of the Society for such purposes as will promote its welfare and that of the profession.

ARTICLE VII.—CHARTER.

The Society shall apply to the State Association for a charter at the meeting at which this constitution and by-laws are adopted, or as soon thereafter as practicable, and the charter shall be kept in the custody of the secretary.

ARTICLE VIII.—TRUSTEES AND INCORPORATION.

The Society shall have authority to appoint a Board of Trustees and to provide for articles of incorporation whenever it may deem the same necessary.

ARTICLE IX.—AMENDMENTS.

The Society may amend any article of this constitution by a two-thirds vote of its members at any regular meeting, provided that such amendment shall have been read in open session at two previous regular meetings and shall have been sent by mail to each member ten days in advance of the meeting at which final action is to be taken.

### BY-LAWS.

### CHAPTER I.—MEMBERSHIP.

SECTION 1. The Society shall judge of the qualification of its members, but, as it is the only door to the State Medical Association and to the American Medical Association for physicians within its jurisdiction, every reputable and legally qualified physician in ———— county who is practicing or who will agree to practice non-sectarian medicine shall be entitled to membership.

SEC. 2. Applications for membership shall be made in such form as may be provided, and all elections for membership shall be by open ballot or by yea and nay vote, and a four-fifths majority of those present shall be necessary for an election.

SEC. 3. Any physician in the county who may feel aggrieved by the action of the Society in refusing him membership, or in suspending or expelling him, shall have the right of appeal to the Council of the State Association.

SEC. 4. Any physician living near the county line, or for other reasons satisfactory to this Society, or upon the decision of the Council of the State Association, upon appeal, may hold his membership in another county society.

SEC. 5. When a member in good standing moves to another county in this or some other State, he shall be entitled to a transfer card to the society into whose jurisdiction he moves, without expense, upon payment of his dues to the date of his removal from this county.

SEC. 6. All members shall be equally privileged to attend all meetings and take part in all proceedings, and shall be eligible to any office or honor within the gift of the Society, so long as they conform to this constitution and by-laws, including the payment of the dues to the Society and to the State Association; provided, that no member under sentence of expulsion shall take part in any of the proceedings, or be eligible to any office until relieved of such disability.

SEC. 7. Kindly efforts in the interest of peace, conciliation or reformation so far as possible and expedient, shall precede the filing of formal charges affecting the character or standing of a member, and the accused shall have opportunity to be heard in his own defense in all trials and proceedings of this nature.

CHAPTER II.—POWERS AND DUTIES.

SECTION 1. This Society shall have general direction of the affairs of the medical profession of the county, and its influence shall be constantly exerted to better the scientific, material and social condition of every physician within its jurisdiction. Systematic efforts shall be made by each member, and by the Society as a whole, to increase the membership until it embraces every reputable physician in the county.

SEC. 2. A meeting shall be held at — p.m. on the — in each month (or oftener). — members shall constitute a quorum. The officers and committee on program shall profit by experience and by the example of other similar societies, and strive to arrange for the most attractive and successful proceedings for each meeting. Younger members especially shall be encouraged to do postgraduate and original research work, and to give this Society the first results of such labors. Crisp papers and discussions and reports of cases shall be arranged for and encouraged, and tedious and profitless proceedings and discussions shall be avoided as far as practicable.

SEC. 3. Agreements and schedules of fees shall not be made by nor in this Society, but at least one meeting during each year shall be set apart for a full and frank discussion of the business affairs of the profession of the county, with the view of adopting the best methods of the most successful members as rules for the guidance of all. In all proper ways the public shall be taught that business methods and prompt collections are essential to the equipment of the modern physician and surgeon, and that it suffers even more than the profession where this is not recognized.

SEC. 5. The Society shall endeavor to educate its members to the belief that the physician should be a leader in his community, in character, in learning, in dignified and manly bearing, and in courteous and open treatment of his brother physicians, to the end that the profession may occupy that place in its own and the public estimation to which it is entitled.

CHAPTER III.—OFFICERS.

SECTION 1. The officers of the Society shall be elected at the (April) meeting each year, which shall be known as the annual meeting. Nominations shall be made by informal ballot, and all elections shall be by ballot. The vote of a majority of all the members present shall be necessary to an election.

SEC. 2. The President shall preside at all meetings of the Society, and perform such other duties as custom and parliamentary usage may require. He shall be the real head of the profession in the county during the year, and it shall be his pride and ambition to leave it in better condition as regards both scientific attainments and harmony than at the beginning of his term of office.

SEC. 3. The Vice-President shall assist the President in the performance of his duties, shall preside in his absence, and upon his death, resignation or removal from the county, shall succeed to the presidency.

SEC. 4. The Secretary shall be the chairman of the Committee on Program and Scientific Work. He shall record the minutes of the meetings and receive and care for all records and papers belonging to the Society, including its charter from the State Association. He shall keep account of and promptly turn over to the Treasurer all funds of the Society which may come into his hands. He shall make and keep a correct list of the members of this Society in good standing, noting of each his correct name, address, place and date of graduation, and the date of the certificate entitling him to practice medicine; and in a separate list he shall note the same facts in regard to each legally qualified physician in this county not a member of this Society, and it shall be his duty to send a copy of such lists upon blank forms furnished him for that purpose, to the Secretary of the State Association, at the close of the annual meeting for the election of officers in each year. In making such lists he shall endeavor to account for each physician who has moved into or out of the county during the year, stating, when possible, both his present and past address. At the same time, and with his report of such lists of members and physicians, he shall transmit to the State Association his order on the Treasurer for the the annual dues of the Society, of two dollars for each member in good standing.

SEC. 5. The Treasurer shall receive all dues and money belonging to the Society from the hands of the Secretary or members, and shall pay out the same only upon the written order of the Secretary.

SEC. 6. The Delegates shall attend and faithfully represent the members of this Society and the profession of this county in the State Association, and shall make a report of the proceedings of that body at the next annual meeting of this Society.

### CHAPTER IV.—COMMITTEE.

There shall be a standing Committee on Program and Scientific Work, of which the Secretary shall be chairman, a Committee on Public Health and Legislation, a Committee on Social Entertainments and Refreshments, each to consist of three members, and such special committees as may be deemed necessary.

CHAPTER V.—FUNDS AND EXPENSES.

The annual dues for each member of this Society shall be (three) dollars, to be paid on or before the annual meeting for the election of officers in each year. (One) dollar of such dues shall be used to defray the expenses of this Society, and two dollars shall be forwarded by the Secretary, with his annual report, to the State Association. Any member who shall fail to pay his dues on or before the date named shall be held as suspended in this Society, and in the State Association, and his name shall be placed on the list of non-affiliated physcians in the report to the State Association for that year, and shall so remain until such disability is removed.

CHAPTER VI.—ORDER OF BUSINESS.

The order of business shall be as follows:

1. Call to order by the President.
2. Reading of minutes of last meeting.
3. Clinical cases.
4. Papers and discussions.
5. Unfinished business.
6. Miscellaneous business.
7. Announcements.
8. Adjournment.

CHAPTER VII.—RULES OF ORDER.

The deliberations of this Society shall be governed by parliamentary usage as contained in Robert's Rules of Order, unless otherwise determined by vote.

CHAPTER VIII.—AMENDMENTS.

These by-laws may be amended at any regular meeting by a two-thirds vote therefor, provided, that such amendment has been read in open session at the preceding regular meeting and a copy of the same has been sent to each member by the Secretary ten days in advance of the meeting at which final action is to be taken.

